# Evidence for Status Epilepticus and Pro-Inflammatory Changes after Intranasal Kainic Acid Administration in Mice

**DOI:** 10.1371/journal.pone.0150793

**Published:** 2016-03-10

**Authors:** Mounira Sabilallah, Pierre Fontanaud, Nathalie Linck, Badreddine Boussadia, Ronan Peyroutou, Thibault Lasgouzes, François A. Rassendren, Nicola Marchi, Helene E. Hirbec

**Affiliations:** 1 CNRS, UMR 5203, Institut de Génomique Fonctionnelle, Montpellier, France; 2 INSERM, U1191, Montpellier, France; 3 Université de Montpellier, UMR5203, Montpellier, France; 4 Labex ICST, Montpellier, France; 5 Plateforme Imagerie du Petit Animal Montpellier, Biocampus, Montpellier, France; University of Modena and Reggio Emilia, ITALY

## Abstract

Kainic acid (KA) is routinely used to elicit status epilepticus (SE) and epileptogenesis. Among the available KA administration protocols, intranasal instillation (IN) remains understudied. Dosages of KA were instilled IN in mice. Racine Scale and Video-EEG were used to assess and quantify SE onset. Time spent in SE and spike activity was quantified for each animal and confirmed by power spectrum analysis. Immunohistochemistry and qPCR were performed to define brain inflammation occurring after SE, including activated microglial phenotypes. Long term video-EEG recording was also performed. Titration of IN KA showed that a dose of 30 mg/kg was associated with low mortality while eliciting SE. IN KA provoked at least one behavioral and electrographic SE in the majority of the mice (>90%). Behavioral and EEG SE were accompanied by a rapid and persistent microglial-astrocytic cell activation and hippocampal neurodegeneration. Specifically, microglial modifications involved both pro- (M1) and anti-inflammatory (M2) genes. Our initial long-term video-EEG exploration conducted using a small cohort of mice indicated the appearance of spike activity or SE. Our study demonstrated that induction of SE is attainable using IN KA in mice. Typical pro-inflammatory brain changes were observed in this model after SE, supporting disease pathophysiology. Our results are in favor of the further development of IN KA as a means to study seizure disorders. A possibility for tailoring this model to drug testing or to study mechanisms of disease is offered.

## Introduction

Animal models of epilepsy are used to elucidate the pathophysiology of seizure activity or to assess the efficacy of anti-epileptic drugs. Among the available models, injection of kainic acid (KA) represents an effective means to elicit status epilepticus (SE) and to induce chronic seizures [1–3]. KA can be delivered in mice using intracerebral (IC) or intraperitoneal (IP) injections. Direct hippocampal KA injection is a surgical procedure resulting in SE and development of focal seizure activity [4]. Conversely, IP KA administration is non-invasive and easy to perform [5]. However, the bioavailability of IP KA is uncontrolled, leading to outcome variability and unpredictable mortality [6]. KA IP re-injections can be performed to ensure SE in all mice [6], potentially introducing a bias deriving from the dissimilar dosages injected.

Drug administration *via* nasal epithelium absorption (intranasally, IN) is pharmacologically recognized, a non-invasive technique, that can be used to deliver drug to the brain [7]. In general, the IN route combines the advantages of the IP and intracerebral delivery, e.g., not surgical and consistent absorption [8,9]. Interestingly, IN administration of KA was shown to provoke acute central cytotoxicity and neuronal damage, reflecting its penetration into the brain [10]. However, it remains unknown whether an appropriate dosage of IN KA may be used to trigger electrographic SE and the associated sequel of pro-inflammatory changes [11].

In the present study we quantified SE onset following IN KA in mice. We also defined the effect of IN KA induced SE on brain inflammation, and microglia and astrocytes activation, including the quantification of M1 and M2 genes using RT-PCR and IBA1/GFAP immunohistochemistry. Our results indicated that SE onset is attainable using IN KA with the technical benefit of low mortality (10–30%) and elevated percentage (>90%) of mice developing SE. Electrographic changes provoked archetypical brain pro-inflammatory signs. A 2 months video-EEG follow up was performed using a limited cohort of mice. Initial evidence prospects seizure progression in this model.

## Materials and Methods

### Animals, seizure induction and behavioral scoring

This study was performed in accordance with the regulations outlined by the French law. The animal experiment protocols used in this study were approved by the Comité d'Ethique pour l'Expérimentation Animale Languedoc Roussillon (CEEA-LR) (N°1142 and 00846.01). All experiments followed European Union (Council directive 86/609EEC) and institutional guidelines for the care and use of laboratory animals. Animals were sacrificed using sodium pentobarbital IP, and all efforts were made to minimize suffering.

Mice were hosted at the IGF animal facility (institutional license approved by the French Ministry of Agriculture N° D34-172-13). Mice were housed on a 12h light-dark cycle with food and water *ad libitum*. We used male C57BL/6J (PN60-70, n = 16) and male C57BL/6J CX3CR1^+/eGFP^ (52 KA injected and 21 sham) adult (PN 60–70) mice [12]. Thirteen mice were implanted with cortical EEG electrodes for video EEG recordings (see below). Out of the 52 KA-injected mice, 6 died due to excessive SE (5 non-implanted and one implanted). The 55 non-implanted surviving mice were divided into experimental groups as followed: 16 Controls; 12 sacrificed 24h after KA treatment; 12 mice sacrificed 72h after KA treatment; 7 mice sacrificed 15 days after KA treatment; 8 mice only processed for behavioral scoring. C57BL/6J CX3CR1^+/eGFP^ mice were used as microglial cells express the green fluorescent protein (eGFP) while retaining normal functional properties [13], therefore amenable to study microglial activation during seizures [6,13,14].

Mice were anesthetized with 2% Isoflurane (Anestéo, France). KA (AbCam, France) was dissolved in NaOH/PBS (10 mg/ml) and instilled in the mouse nostrils [10]. Mice were maintained under isofluorane anesthesia during KA administration. Ten μL of KA (30 mg/kg) was delivered (alternate in each nostril) every 2 minutes for a total of 70–90 μL. Each administration (10 μL) lasted 10–15 seconds and did not interfere with mouse anesthesia. Sham mice received IN PBS. All mice woke up within 5 minutes following the final IN administration. IN PBS did not induce behavioral or histological changes (*data not shown*). After IN KA, behavioral changes were monitored using the Racine Scale [6,15]. Stage-1: freezing behavior; Stage-2: rigid posture and tail; Stage-3: head nodding, rearing into a sitting position with forepaws shaking; Stage-4: rearing and occasional falling accompanied by period of stillness; Stage-5: bilateral forelimb clonus with rearing and loss of balance; Stage-6: repetitive loss of balance and tonic-clonic activity. Mouse behavior was scored for 2h every 15 min; the eight values were used to calculate a “*mean score*”. Mice were sacrificed (24h, 72h or 15 days post-SE) using sodium pentobarbital administration and transcardiacally perfused with 10ml RNAse free PBS. Brains were removed and divided into two parts along the sagittal axis. One half was fixed in 4% paraformaldehyde for 24 hours. Brains were then placed in PBS solution containing 0.1% sodium azide. The other half was dissected to isolate the hippocampus, snap frozen and stored at -80°C.

### Video- EEG recording and analyses

Thirteen mice (5 sham and 8 IN KA) were implanted with cortical EEG electrodes. Briefly, mice were anesthetized with ketamine/xylazine, placed on a stereotaxic frame and the skull exposed. Four stainless steel screws were placed bilaterally on the *duramater* of the fronto-parietal cortex. A prefabricated pre-amplifier (2 differential channels, Pinnacle Inc., USA) was connected to the cranial screws. The assembly was then sealed with dental acrylic resin. Mice were left unrestrained for one week. The implanted mice were recorded before KA-administration (baseline), during SE (0-4h), during the 72 hours post-SE, and every other day starting from 15 days post-SE and up to approximately 2 months. Except for the recordings performed immediately after KA-administration all recordings were performed overnight for 7 hours, during the activity period (10:00 pm to 5:00 am). EEG signals were acquired at 200Hz using band pass filter (50Hz) and were analyzed using a combination of commercially available software (p-Clamp 9.2; Origin Microcal) and a routine (Matlab 2013b; Matworks Inc.) optimized in our laboratory. Sham animals (IN PBS) displayed no EEG changes. Acute SE was characterized by generalized EEG convulsions (on/off episodes lasting >2 minute in the acute phase) developing to synchronized continuous generalized activity 45 to 60 minutes after. Spike activity was defined as symmetrical EEG signals with amplitude of at least 2–3 fold the baseline. In the chronic phase SE was characterized by generalized EEG activity lasting at least 10–15 sec. Video review was performed to rule out EEG artifacts (scratching, chewing or sleep). For each mouse we determined the time spent in spike activity and SE during seizure progression. EEG data were obtained as X/Y (msec/ V) Excel or European Data Format (.edf) files. We applied a Short Time Fourier Transform to the EEG files calculating signal energy as a function of frequency and time. Power spectra were estimated as the square of the Fourier transform for each period analyzed.

$$STFT\left(\omega ,t\right)=\int_{-\infty }^{\infty }eeg\left(t\right)\;\psi \left(t-\tau \right)e^{-i\omega t}dt$$ (1)

In particular, ψ(t) = Bartlett function; ω = frequency in hertz; τ = translation time form segment to segment; t = time in seconds; eeg (t) = EEG signal. A qualitative measure for EEG frequency bands (δ, θ, α, β, γ) was performed. The latter measurement is defined as:

$$\int_{\omega_{min}^{i}}^{\omega_{max}^{i}}I\left(\omega ,t\right)\;d\omega \;\;i=\delta ,\theta ,\alpha ,\beta ,\gamma$$ (2)

Where ($\omega_{min}^{i}$,$\omega_{max}^{i}$) are the frequency limits for the band I and I(ω,t) is the power spectrum as a function of frequency and time t.

Out of the 8 implanted and KA-treated mice, one died early after the KA administration due to intense SE. Another mouse was excluded from the study as recordings regularly failed due to technical reasons. The 6 KA-treated mice included in the study were scored using the Racine Scale at *posteriori* by analyzing of the video recording.

### RNA Extraction and Quantitative PCR

Total RNA was extracted from whole hippocampi using the RNeasy Plus universal midi kit (Qiagen) 24h and 72h after induction of SE. The quality control of RNA was performed using the Agilent 2100 Bioanalyzer (Agilent). All RNAs used had RNA Integrity Numbers (RINs) above 8.0. Between 0.5–1 μg of total RNA were reverse transcribed using the iScript kit (BioRad). RT products were diluted 10 times with H_2_O and stored at -20°C until use. Real-time PCR was performed in 384 well plates in a final volume of 10 μl using SYBR Green dye detection on the LightCycler480 system (Roche-Diagnostic). Genes analyzed were general markers of neuroinflammation (Gfap; Timp1, Cxcl10, Cox2, Igf1); markers of quiescent microglia (P2rY12), and markers of M1 (Il1β, Fcgr1, Tnfα) or M2 (Tgfβ, Ccl2, Gal3) microglia phenotype. The primer pairs were either commercially available (Quantitec primers, Qiagen) or designed using Primer 3 input software, and their specificity and efficacy was experimentally validated. Cq for individual genes were determined using the second Derivative Max tool of the LightCycler480 software. The relative RNA expression was calculated using Gapdh as a reference gene. Indeed there was no statistical variation in the level of expression of Gapdh across the experimental groups. Relative expression between KA-treated and control groups were calculated using the ΔΔCq method [16]. For genes undetectable under control conditions, the fold change (FC) increase was determined by attributing a Cq value of 35 corresponding to the maximal value in our experimental conditions. Thus, under these conditions FC values were underestimated.

### Immunohistochemistry and quantifications

Fixed brains were cut into 40 μm sections using a Vibratome (Microm, France). Sections were permeabilized with 0.1% Triton X-100 in PBS, 2% Bovine Serum Albumin (BSA) and then incubated for 48h at 4°C with primary antibody (anti-IBA1 1:2000, Wako; anti-GFAP 1:500, Dako). After three washes in PBS, sections were incubated with the appropriate secondary antibody. Wide field or Apotome images of the whole unilateral hippocampus were acquired using an Imager Z1 microscope (Zeiss) equipped with an AxioCam MR3 camera. For analysis of IBA1 staining, for each field, 11 images corresponding to 10 μm-thick optical sections were acquired. For analysis of GFAP staining, for each field, 5 images corresponding to 4 μm-thick optical sections were acquired. Stacked images were merged and analyzed through the use of a custom developed ImageJ macro to determine (1) the number of immunopositive cells and mean cell body size (IBA1 staining) and (2) the percentage of immunopositive cell surface (GFAP staining). Fluoro-jade staining was performed according to manufacturer recommendations. Slices were examined with Imager Z1 microscope (Zeiss). The presence of positively stained neurons was observed in 6 sections randomly selected from the rostral and caudal portions of the hippocampus.

### Statistics

Quantifications were performed in at least 5 animals per experimental condition. The number of animals included in the different analyses is provided in Table 1. For qPCR experiments, Mann-Whitney non-parametric tests were applied at each time point to test significance between the treated group and the control group. For immunohistochemistry experiments, quantifications were performed in 4 randomly selected fields per section and 5 sections per animal. Kruskall-Wallis one-way analysis of variance test followed by Dunn’s post-test was applied to test significance between groups.

**Table 1:** Number of mice included in the different analyses (stage≥5 Racine).

	qPCR	IBA1 staining	GFAP Staining
**24h**			
** Control**	12	15	9
** KA-treated**	7*	8	8
**72h**			
** Control**	6	same as 24h	same as 24h
** KA-treated**	10	10	9*
**15D**			
** Control**	ND	same as 24h	same as 24h
** KA-treated**	ND	5	5
**49D**			
** Control**	ND	5	5
** KA-treated**	ND	7	7

*: one sample missing due to technical issues during the processing

ND: Not determined

## Results

### Acute Behavioral and EEG changes after IN KA

**Fig 1:**
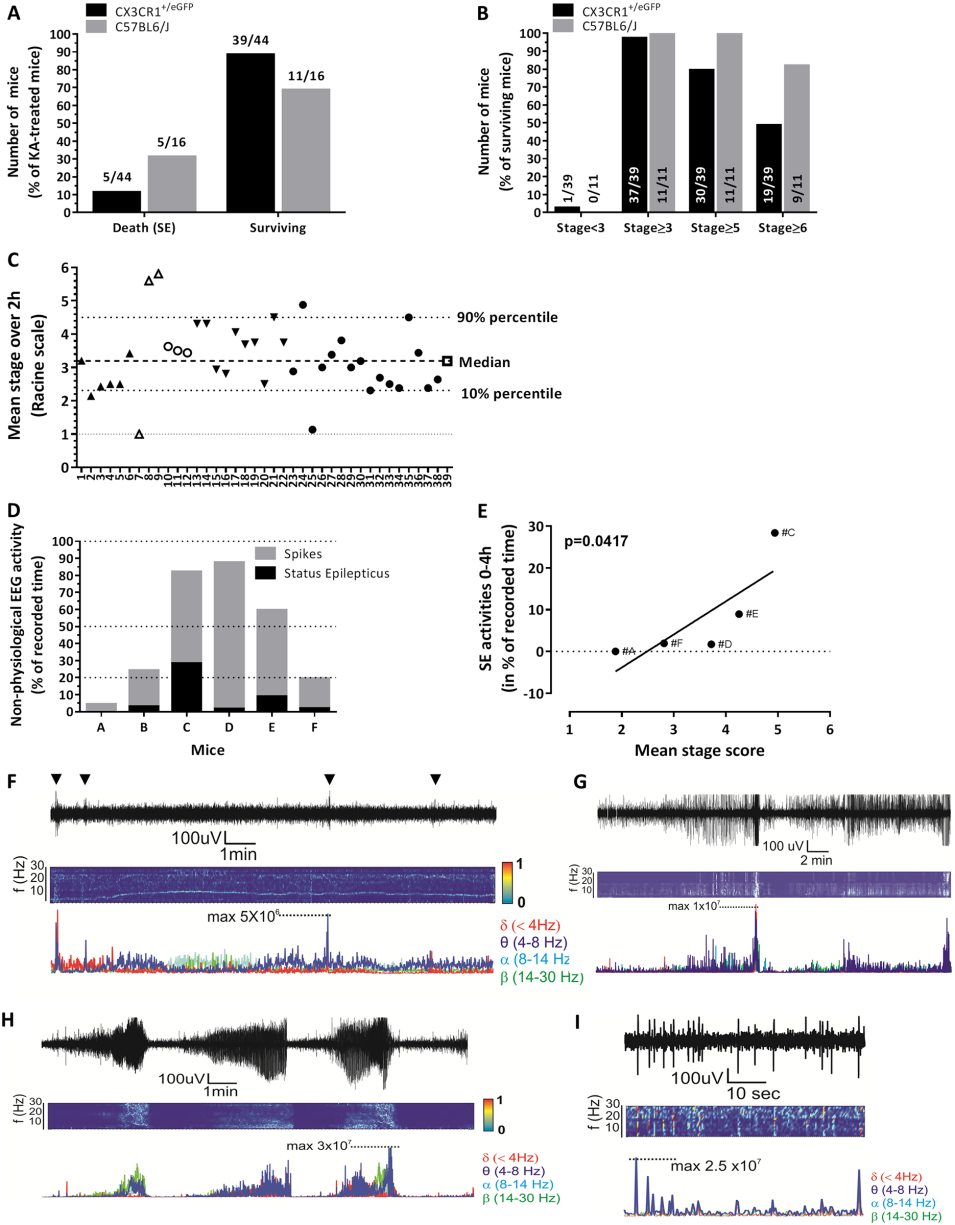
Behavioural and video-EEG read-outs after IN KA. **A)** Mortality after SE induced by IN KA administration in C57BL6/J and C57BL6/J CX3CR1^+/eGFP^ mice. **B)** Distribution of Racine scores in C57BL6/J and C57BL6/J CX3CR1^+/eGFP^ mice. The majority of surviving mice reached stages 6 (>80% in C57BL6/J and ≈50% in C57BL6/J CX3CR1^+/eGFP^). Values indicate the number of mice at each Racine stage. **C)** Racine mean score over the 2 hours following IN KA. Identical symbols indicate mice assessed on the same day. **D)** Video-EEG was used to quantify duration of SE (black bars) and spike activity (grey bars) within the 4 hours following IN KA. **E)** Correlation between the Racine mean score and the time spent in EEG SE. **(F-I)** Examples of EEG recordings and analysis during baseline activity (F), status epilepticus (G-H) and spike activity (I). Time joint-frequency analysis and single frequency profile are also provided.

IN KA > 45 mg/kg in C57BL/6J mice provoked high mortality (100%, *data not shown*), therefore setting an upper limit to the IN protocol. At 30 mg/kg IN KA dose, the mortality rate was of 30% (5/16), with 93% (15/16) of animals experiencing ≥ Stage 5 in the 2 hours post-KA (Fig 1A and 1B). In C57BL/6J CX3CR1^eGFP/+^ mice [6], the mortality associated with SE after IN KA was reduced to 12% (5 out of 44, Fig 1A) while the rest of the mice (n = 39) experienced Stage 3 to Stage 6 Racine behavioral changes (Fig 1B). Thirty-one mice reached Stage 5 while 19 mice further progressed to Stage 6 experiencing tonic-clinic seizures (Fig 1B). Fig 1C indicates the mean Racine score calculated throughout 2 hours of observation for each mouse (see Materials and Methods). In particular, 80% of the surviving mice exhibited a mean score between 2.3 and 4.5 Racine. Two mice experienced repetitive Stage 6. The score was independent of the experiment or experimenter.

Video-EEG was performed to further characterize SE onset and seizure progression after IN KA. Fig 1D summarizes the percentage of cumulative time spent in SE and abnormal spike activity during the 4 hours following IN KA. Video monitoring was used to confirm Racine changes concomitant to EEG patterns, (i.e. EEG SE corresponded to Racine 5–6). Fig 1E shows the significant correlation between the mean stage scores (behavior) and time spent in SE, as recorded using EEG. Examples of EEG recordings and time-joint frequency analysis are provided in Fig 1F–1I. Spike activity was recorded up to 36 hours after IN KA in mouse #C, the latter having experienced repetitive SE during the acute phase (see Fig 1D). Return to baseline activity occurred during the 72 hours following IN KA (*data not shown*).

### Hippocampal neurodegeneration and transcript levels of inflammatory mediators after IN KA

**Fig 2:**
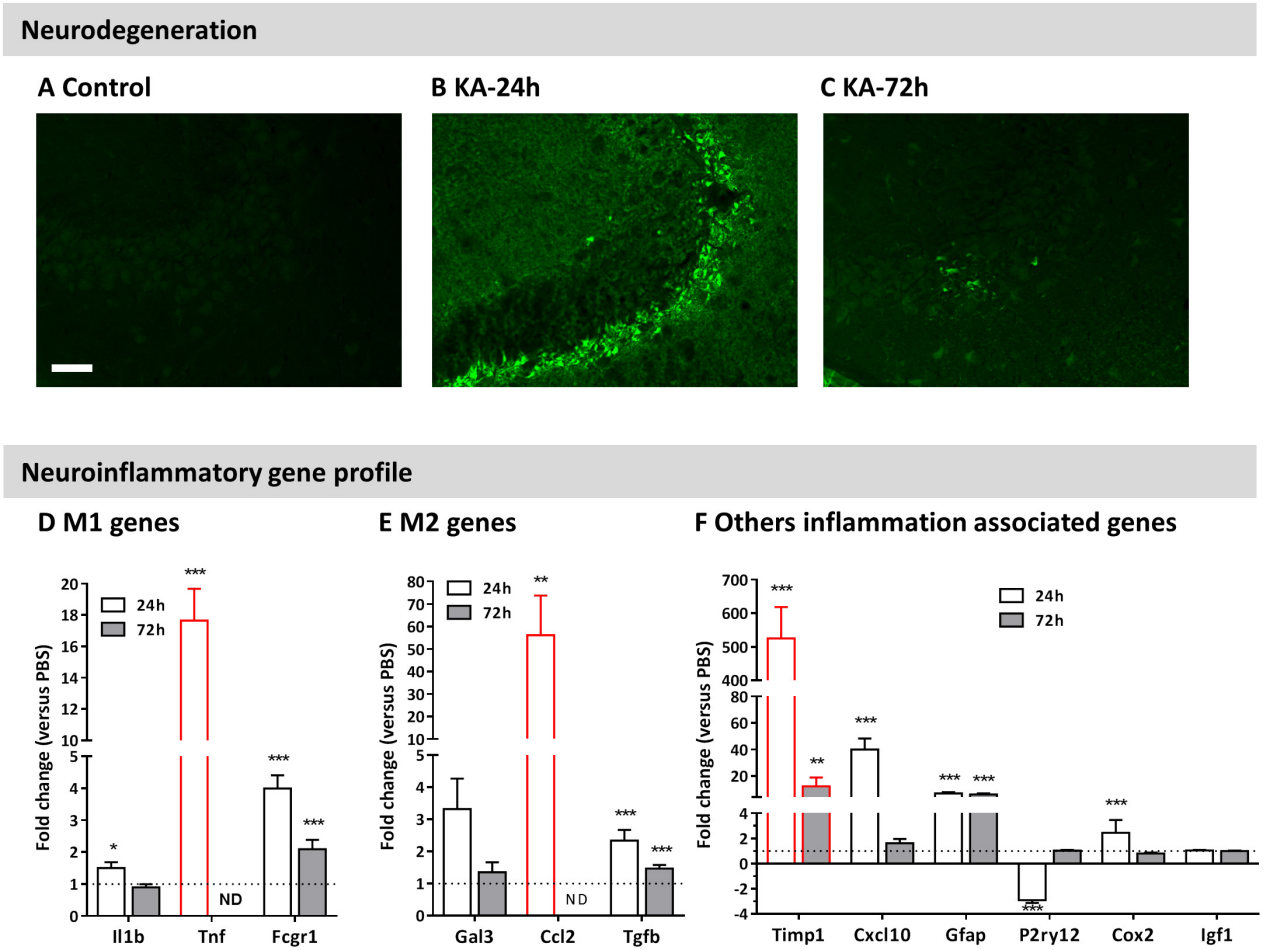
Neurodegeneration and neuroinflammatory gene profile after IN KA induced seizures. **A-C)** Fluoro-Jade C staining is observed 24h after IN KA, diminishing at 72h. Correspondence existed between presence of FJC positive neurons and behavioral score after IN KA (B: Mouse 9, mean-score = 5.8; C: Mouse 28, mean-score = 3.8). Scale bar 50 μm. **D-F)** qPCR analysis and changes of inflammatory gene levels in mice experiencing stage 5 or higher. Analysis was performed 24h (white bars) and 72h (grey bars) after IN KA. Panels (D) and (E) represent mRNA changes for M1 and M2 microglial markers. Red bars indicate genes that are not detectable under control conditions (see materials and methods section). Results are presented as mean ± SEM (n ≥ 7 / group). Statistical analysis was performed using a non-parametric Mann-Whitney test between control and KA-treated conditions. *: p<0.05; ** p<0.01; ***: p<0.001 compared to controls.

Acute neuronal degeneration was assessed using FluoroJade C (FJC) staining 24h and 72h following IN KA (Fig 2A–2C). At 24h, FJC-positive neurons were observed mostly in the CA3 and, occasionally CA1 regions. Animals presenting a 2h mean-stage-score >3.5 presented FJC positive neurons (Fig 2B). Three days following SE, only sparse FJC-positive neurons were detected (Fig 2C). We then evaluated whether IN KA induced SE is associated with inflammatory changes in the hippocampus. Animals exhibiting at least a Racine stage 5 were included for analysis. We used qPCR to quantify the transcriptional remodeling of a series of genes, including M1 (Il1β, Fcgr1, Tnfα), M2 (Tgfβ, Ccl2, Gal3) selective microglial phenotype markers and inflammatory markers (Timp1; Cox2; Igf1, Cxcl10; Gfap) [17]. Gene levels (with the exception of Igf1), were significantly changed 24 hours post SE as compared to control (fold changes; Fig 2D–2F). Seventy-two hours following SE, the expression of the majority of the genes returned to control values, with the exception of Fcgr1 and Tgfß that remained significantly elevated. Genes associated to neuro-inflammatory changes (Timp1 and Gfap) were up-regulated at 72h (Fig 2F). These results show the pathological effects of SE induced by IN KA.

### Astrocytes and microglia activation after IN-KA administration

**Fig 3:**
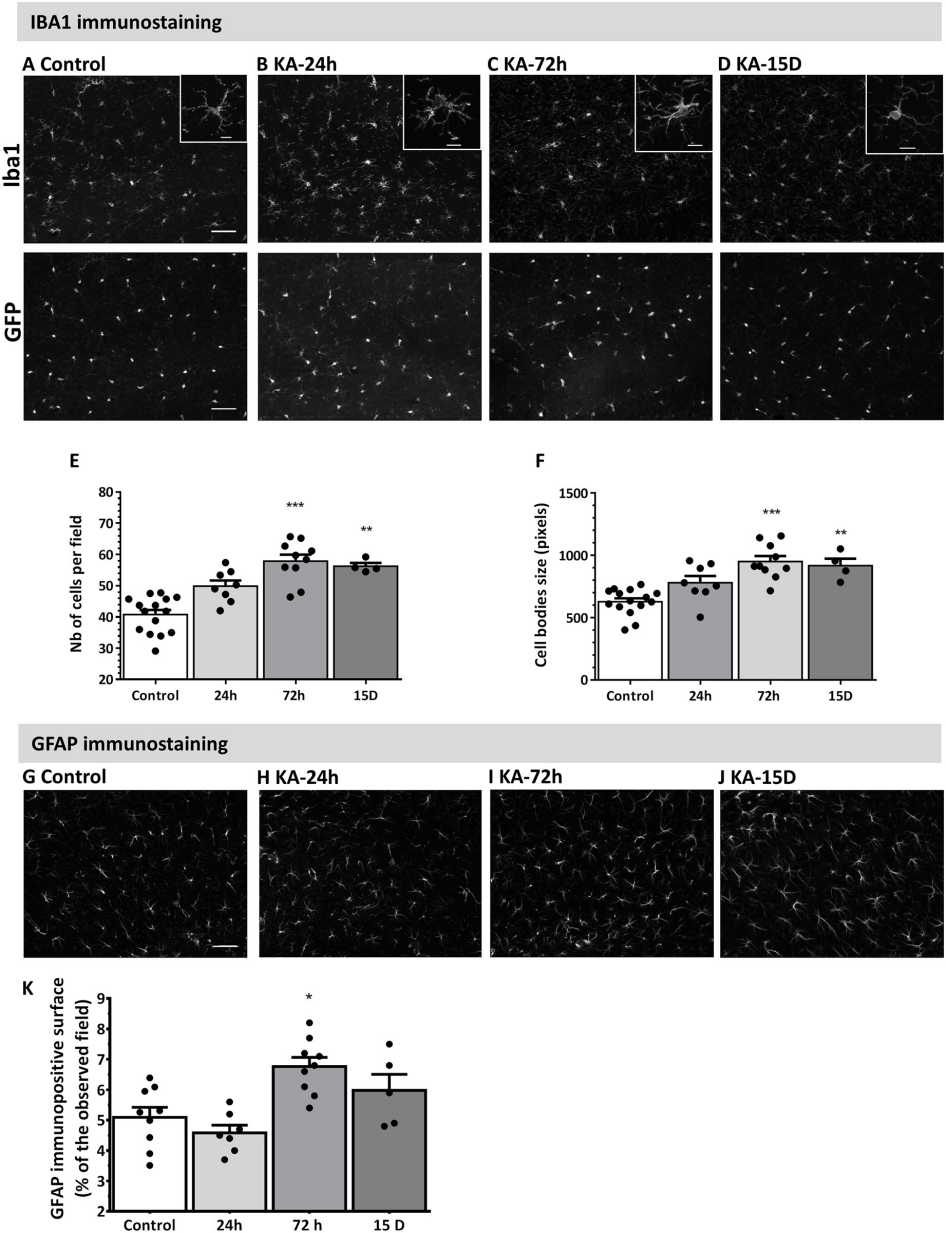
Time-dependent microgliosis and astrogliosis following IN KA induced seizures. Analysis performed in mice experiencing stage 5 or higher. **A-D)** Representative images of IBA1 immunostaining (upper panels) and GFP fluorescence (C57BL6/J CX3CR1^+/eGFP^ mice; lower panels) in CA1 region in control mice (A), and 24h (B), 72h (C) and 15 days (D) after IN KA. Scale bar = 50 μm. Insets depict enlarged image of individual microglial cells. Scale bar = 10 μm **E-F)** Quantitative analysis of IBA1 immunostaining in the hippocampus 24h, 72h and 15 days after IN KA-induced SE shows significant microglial activation, including increased cell number and size. **G-I)** Representative images of GFAP immunostaining in CA1 region in control mice (G), 72h (H) and 15 days (I) after IN KA. Scale bar = 20 μm. **J)** Quantitative analysis of GFAP immunostaining in the hippocampus shows astrogliosis 72h after IN KA. Results are represented as mean ± SEM (n ≥ 6 per group). Statistical analysis was performed using a non-parametric Kruskal-Wallis one-way analysis of variance followed by Dunn’s post-test. *: p<0.05; **: p<0.01; ***: p<0.001 compared to control condition.

Hippocampal microgliosis and astrogliosis were investigated following IN KA using endogenous CX3CR1^eGFP/+^ microglial fluorescence, and GFAP or IBA1 immunostaining (Fig 3A and 3G) [18,19]. Time-dependent microglial activation, characterized by hyperplasia and hypertrophy, was detected starting from 24h post SE (p>0.05), peaked at 3 days and persisted for 15 days (p<0.01; Fig 3E and 3F). IN KA also elicited significant hippocampal astrogliosis 3 days post SE (Fig 3G–3J), subsequently decreasing 15 days post SE.

### Initial evidence for seizure progression after IN KA

**Fig 4:**
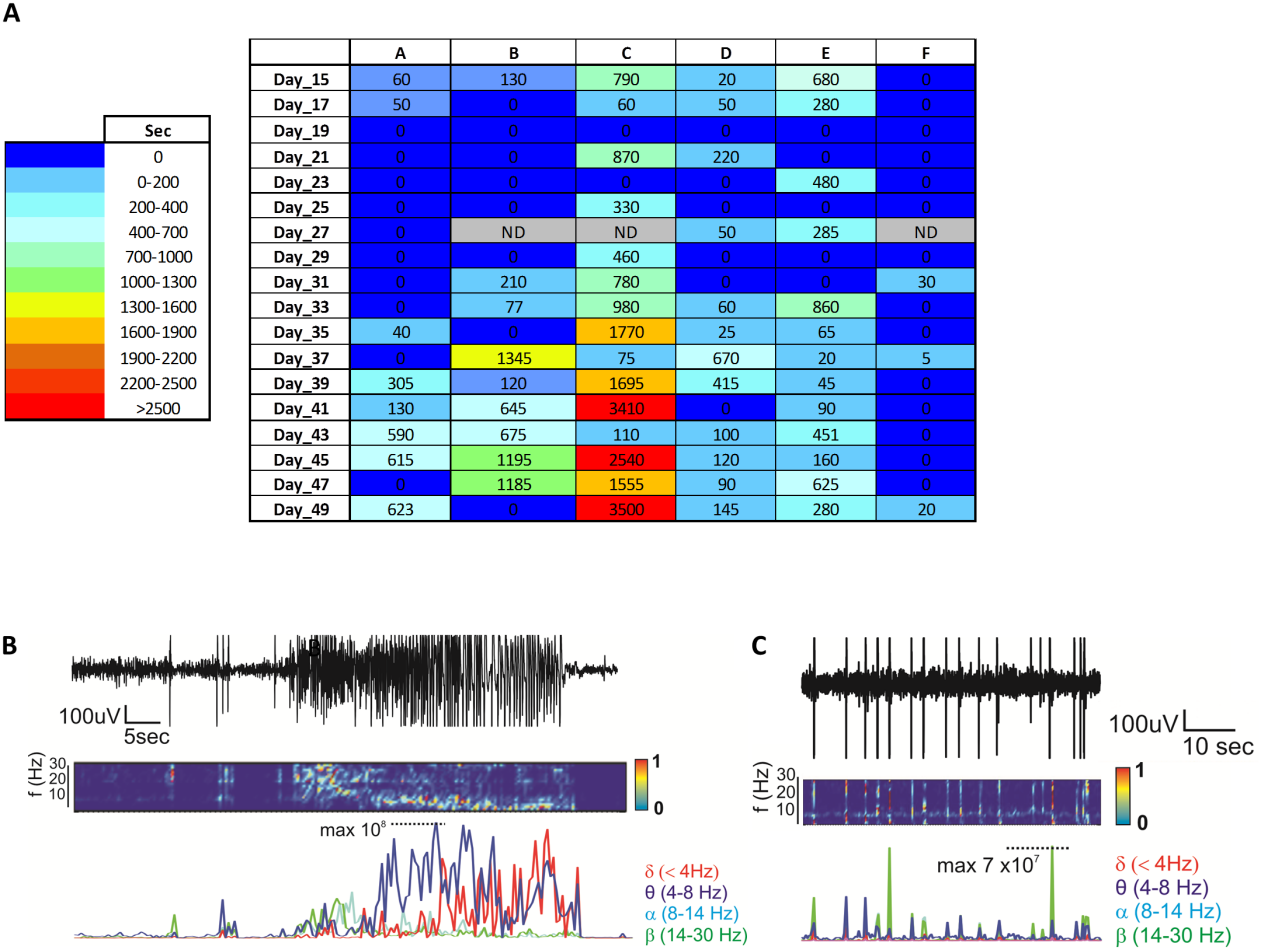
Video-EEG analysis during seizure progression. **A)** Heat map (blue to red) and raw numbers indicate the sum of durations (in seconds) of spike activity recorded for each animal in the given recording session. **B-C)** Examples of EEG recordings during the chronic phase (*e*.*g*., SE in B and spike activity in C).

**Fig 5:**
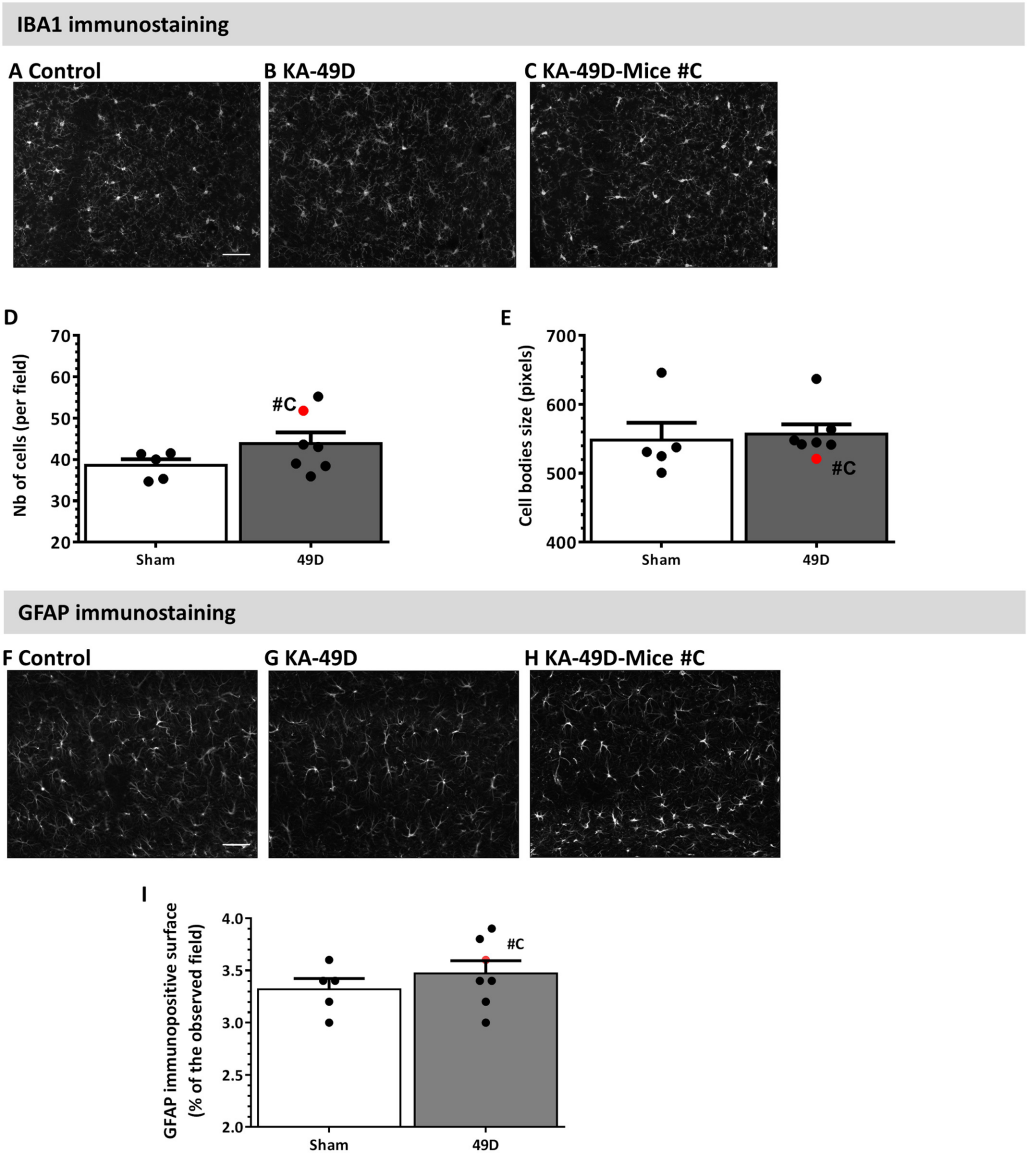
Lack of long-term microglia and astrocytes reactivity. **A-C)** Representative images of IBA1 immunostaining in CA1 region in control implanted mice, IN KA mice and mouse #C (see below). Scale bar = 50 μm. **D-E)** IBA1 analysis shows no significant microglial activation in KA mice compared to control mice. Mouse #C experienced severe SE in and displayed elevated microglia density (*red data* in D). **F-H)** Representative images of GFAP immunostaining in control implanted mice, KA- mice and mouse C. Scale bar = 50 μm. **I)** Quantitative analysis of GFAP immunostaining in the hippocampus shows no difference at 49 days post KA. Results are presented as mean ± SEM. Statistical analyses were performed using a non-parametric Mann-Whitney test.

A longitudinal Video-EEG monitoring was performed using our small cohort of mice (see Material and Methods). The sum of spike activity durations (seconds) was quantified for each recording sessions and indicated as a heat-map for each mouse (Fig 4A). With the exception of mouse #F, spike activity increased in all mice from 30 days post IN KA. Example of spike activity is shown in Fig 4C. Remarkably, mouse# C experienced three SE (days 39, 41 and 47; Fig 2B), also confirmed by video examination. No SE was recorded in the rest of the mice using our EEG protocol (see Methods). Microglia activation and reactive astrogliosis were also investigated at the end of the EEG recordings, i.e. 49 days post IN KA. No histological signs of microglial activation were detected, as neither cell number nor size was different from control mice (Fig 5).

## Discussion

Our results indicate that IN KA can be used to elicit acute SE in mice. They show that a dose of 30 mg/kg is associated with low SE-induced mortality and a high yield of mice developing acute SE (>80%), as compared to intra-peritoneal injections where a significant percentage of mice do not survive or do not experience SE [20]. We found IN KA dose > 45 mg/kg to be lethal when using C57BL/6J mice.

Inflammation is one of the hallmarks of epilepsy [21,22]. Inflammation is consequent to acute SE, while a lasting inflammatory response is thought to contribute to epileptogenesis [23,24]. Our data indicate pro-inflammatory changes resulting from IN KA-induced SE, confirming disease pathophysiology. We found phenotypic microglial activation and astrogliosis, along with transcriptional modifications of inflammatory genes. Microgliosis was observed as early as 24 hours after IN KA induced SE. Our results show that both microglial M1 and M2 genes are dysregulated following IN KA induced SE. M1 corresponds to a pro-inflammatory status, correlating to or facilitating neuronal damage. In contrast, the anti-inflammatory M2 phenotype may be beneficial following brain pathological events [25]. Although, we cannot rule out that some of these genes may also be expressed by activated astrocytes, and the number of genes analyzed is limited, our results suggest complex microglia activation following IN KA SE. Our data are consistent with earlier studies performed using 20–40 mg/kg IP KA [6,14]. Remarkably, inflammatory signs were present 15 days after SE onset. Although significant, the inflammatory changes developing after IN KA induced SE were less prominent as compared to the IP protocols, suggesting that the IN KA dose can be still modified to induce a more severe phenotype. In this regard, our results indicate an upper limit for IN KA dosages of 45 mg/kg. While inflammatory changes were evaluated, other pathological hallmark of SE (i.e. cerebrovascular changes) were not evaluated in this study [26–28].

### Initial indication for long-term electrographic changes following IN KA

A key requirement to endorse IN KA as a model to study seizure progression and long-term pathophysiology is to demonstrate longitudinal EEG changes. While 30 mg/kg IN KA was adequate to elicit SE, progression to spontaneous and generalized SE was, in our condition, only partial. We found spontaneous spike activity but only sporadic SE (Fig 4). In addition, duration of spontaneous spike activity varied among mice (Fig 4A). Limitations of our study include the lack of continuous 24/7 video-EEG and the use of intra-hippocampal electrodes. Higher IN KA dosages, between 30 and 40 mg/kg, remain to be tested.

### Technical remarks

The results presented herein support the further development of IN KA administration as a model of seizure progression and pathophysiology. From a technical stand point, the intranasal route is *non*-invasive allowing for a controlled KA delivery. These are combined advantages as compared to IP and IC injections. Recovery from gaseous anesthesia is rapid and does not interfere with behavioral testing or EEG recording. As the mice are anesthetized during IN delivery, stress is greatly reduced. IN KA could thus provide better compliance to animal safety and 3R’s rule, possibly reducing the number of animals used.

Available evidence demonstrates outcome variability to KA across mouse strains [20]. In our study we used C57BL/6J mice or C57BL/6J CX3CR1^+/eGFP^ mice. Inter-studies variability also exists when using KA, depending on strain or sub-strain specificity, environmental factors or intrinsic variability associated with the injection procedures [6,14]. Our results back reproducibility of the IN KA protocol in two mouse lines and demonstrate compatibility of the IN KA protocol with the KA-resistant C57BL/6J background. In addition, SE outcome was independent from the time of the experiment or the personnel performing the administration, showing technical reproducibility. Importantly, reproducibility after single dose administration is adequate, thus facilitating the comparison of KA effects in mice with different genetic backgrounds. In conclusion, while IN KA elicits acute SE and inflammatory changes, the optimal dosages to obtain a robust progression to spontaneous seizures remain to be optimized.
